# In pursuit of community engagement: Exploring its intersections with teaching and learning in emergency care education

**DOI:** 10.4102/hsag.v30i0.3113

**Published:** 2025-12-02

**Authors:** Kubendhren Moodley, Raisuyah Bhagwan

**Affiliations:** 1 Department of Health, College of Emergency Care, Western Cape Government, Cape Town, South Africa; 2 Department of Community Health Studies, Faculty of Health Sciences, Durban University of Technology, Durban, South Africa

**Keywords:** community engagement, emergency medical care, education, higher education, health sciences

## Abstract

**Background:**

Community engagement has gathered attention, as a salient mechanism to improve patient care and healthcare systems, while strengthening students’ awareness of societal ills.

**Aim:**

This article explores the views of healthcare academics in the Western Cape, with regard to the potential of the role of community engagement, in enhancing teaching and learning, in emergency medical care education.

**Setting:**

This study was undertaken in the Western Cape province of South Africa.

**Methods:**

The study adopted qualitative methodology. Two samples of academics from various health science disciplines were recruited and the third sample from emergency medical care specifically, were recruited using non-probability sampling strategies. Data were collected using interviews and focus group discussions with 29 academics in total. Following a process of thematic analysis, the study found that engagement remained at a disjuncture from teaching and learning in paramedic education.

**Results:**

The data revealed that there was huge potential for paramedic students to acquire valuable knowledge, if they were immersed within community spaces as part of work integrated learning. Service learning, volunteerism and outreach were further identified as potential pathways to forming collaborative partnerships that could ensure that emergency medical care graduates were better prepared to understand health disparities and work towards healthier communities in South Africa.

**Conclusion:**

The study advances the need for emergency medical care to extend preparedness for clinical practice through conscientising students about health disparities and to working with vulnerable communities to find solutions to health problems.

**Contribution:**

The study emphasises the importance of integrating community engagement into emergency medical care education.

## Introduction

Community engagement is underpinned by the mandate of the university to improve ‘the lives of communities for the common good through civic engagement’. It has been described as the collaborations and partnerships between the university and the communities it serves, with the intent to build and exchange knowledge, skills, expertise and the resources required to develop society (Preece 2017). Tolken and McKay (2019) added that volunteerism, community outreach, internships, co-operative education, and service learning are typologies for student community engagement. Engagement can therefore entrench itself in higher education, through service learning, community-based research, community-based education and health promotion (Wood 2017).

Although community engagement has been identified as one of the major criteria for the quality assurance of the higher education sector in South Africa, to prepare students for responsible citizenship, it has remained at a disjuncture from emergency medical care education (Govender & Wait 2017). While a large body of research, related to the relevance and impact of community engagement has emerged within many disciplinary homes internationally (London et al. 2024; Maistry 2023; Van Eeden, Eloff & Dippenaar 2021), however, empirical work within emergency medical care specifically is lacking.

Mtawa, Fongwa and Wilson-Strydom (2021) argued that many South African universities have begun to adopt service learning as a mechanism, to promote community engagement and develop socially responsible students. They opined that South African universities have begun to adopt community engagement, to educate students with curriculum-specific knowledge and skills and to teach them citizenship with the intent of developing socially responsible graduates (Bastable et al. 2019). This has relevance for emergency medical care graduates, who should not just be prepared clinically for emergency care, but should also be conscientised with regard to aspects of social responsibility. Koekkoek, Van Ham and Kleinhans (2021) argued that ‘the inextricably contextual aspect of interaction’, positions emergency medical care practitioners, in an ideal space within communities, to engage with and work collaboratively with them, with regard to health care issues, particularly in developing contexts such as South Africa. It is against this backdrop that this article makes a case for emergency medical care education to prioritise community engagement, by exploring the views of academics concerning the transformative potential of community-based teaching and learning.

Community outreach involves student activities where the primary beneficiary is the community and where the goal is to provide a service, which is initiated by the university in a structured manner. Outreach activities are powerful support services, by virtue of their interaction with vulnerable, disadvantaged, and difficult-to-reach communities (Butcher 2017). Whowell (2016) added that outreach delivers services to communities, which lack access to essential services within their geographic location.

Service-learning has also been supported as a salient mechanism to promote engagement because of its ability to interweave the academic activities of modules, with increased participation in a formal intellectual dialogue on service problems (Higher Education Quality Committee [HEQC] 2006a, 2006b). Within the context of service learning, the ‘community’ refers to the distinct, local, collective interest groups, which collaborate within the institution’s service-learning activities. Local communities are no longer viewed as beneficiaries, but rather as participants, who have a voice in identifying service needs and development challenges. Communities therefore participate in establishing service learning and development outcomes, by identifying the appropriate assets they possess, evaluating its effect, and making meaningful contributions to the mutual quest for sustainable solutions to complex problems (Higher Education Quality Committee [HEQC] 2006b)

Huda et al. (2018) added that the strength of service-learning programmes is linked to problem-based learning, collaborative learning, undergraduate research, critical thinking, multiculturalism and diversity, community awareness, leadership abilities, and social and professional responsibilities. These are all important aspects for the robust development of paramedic students’ graduate attributes. Bringle, Hatcher and Jones (eds. 2023) opined that service-learning activities can be used to enhance the achievement of learning outcomes and supplement learning resources, which can be easily integrated, to strengthen the paramedic student’s learning experience. Given that student learning emerges through activities, experiences, strategies for learning, and assessment, there should be a clear link between the objectives of the module and its service activities (Bringle et al. 2023). Through deliberately integrating it into emergency medical care modules in this way, much can be done to advance engagement.

Graduate attributes can therefore be nurtured through focusing on patient-centredness, social responsibility, and sensitisation to social injustices within the South African health care system in emergency medical care education (Van Huyssteen & Bheekie 2013). Some studies have revealed its potential to be integrated into paramedic education. For example, as part of outreach activities, students in the Western Cape, provided health services to underprivileged populations at public facilities, using their knowledge, skills, and medical expertise. Similar strategies were used in other areas of the Western Cape, with First Aid Responders, undertaking their training in marginalised communities, with poor access to emergency care (Sun & Wallis 2012). Despite the value of these immersive experiences for students, engagement remains to be formalised into Diploma in Emergency Medical Care (DEMC) paramedic training as part of the credits towards the qualification. Swanberg et al. (2018) therefore argued that outreach initiatives, in health science faculties, have an invaluable role to play in their surrounding communities, through amplifying health promotion and health education.

Following the globalisation of paramedicine as a profession and the emphasis on outward-bound learning experiences in higher education, Shannon et al. (2023) argued that community engagement should be incorporated into paramedicine curricula at a global level. A study by Simpson et al. (2016), explored the experiences of 12 paramedic students, who participated in their first international work integrated learning placement in South Africa and New Zealand. They documented the importance of these placements, in strengthening experience, but recommended that students be prepared and supported for community engagement or work integrated learning practice in paramedic education (Simpson et al. 2016).

Nicholas (2019) further argued that community engagement was gaining traction because of its ability to improve graduate skills, qualities, and employability, thereby strengthening the rationale for its consideration in paramedic education. Hardie, Almeida and Ross (2018) asserted that through service learning, students can apply theoretical and practical knowledge acquired at university, within a practice context. Students are given the opportunity to learn and integrate academic information, skills, and experiences in a community context, which is relevant to their courses. This type of experiential learning consequently enables students to have direct interaction with community issues (Shelton 2016). Although paramedic students often work in frontline clinical settings, real-world experiences provide richer opportunities for them to be involved in health education and promotion and for partnering with communities to find solutions to health issues. Given the growing body of research on community engagement, it was important to explore the views of academics in health sciences and academics in emergency medical care, with regard to their experiences of community engagement and to understand how they have integrated it, within the context of teaching, learning and research. Therefore, to deeply understand the connection between community engagement and emergency medical care, the study explored the views of academics in health sciences at higher education institutions in the Western Cape, South Africa.

### Theoretical framework

Boyer’s (2016) model on the scholarship of engagement was found to be most appropriate, as the guiding theoretical framework of this study. It emphasises the importance of collaboration and partnership between universities and communities to tackle real-world issues and generate new knowledge, while fostering mutually beneficial relationships (Boyer 2016). His model is underpinned by four components, namely discovery, teaching, application, and integration, which collectively acts as a synergistic whole. Through his model academic disciplinary departments, such as emergency medical care, have been invited to integrate the four scholarships of discovery, integration, sharing and the application of knowledge into their education. Boyer (2016) urged academic departments to consider how knowledge could evolve by harnessing the potential of multiple sets of health disciplines (integration), by continuously validating discoveries with students (sharing) and by generating new knowledge for the real world through application. The scholarship of engagement is therefore predicated on the notion that highly collaborative partnerships outside the universities can generate mutually beneficial benefits not just for students, but academics and community members. This study was appropriate as the guiding theoretical framework, as it reflected how academics, students and communities shared their knowledge through mutually beneficial reciprocal relationships, enriching the learning opportunities for students, while creating enhanced opportunities for communities to benefit from the engagement activities and projects being advanced by health science disciplinary departments.

## Research methods and design

### Study design

Qualitative research methodology was used to guide this study, because of its naturalistic nature and its focus on the experiences of participants (Lim 2024). Given that the researcher sought to uncover thick and rich descriptive data, to develop an understanding of how community engagement, can be enhanced in emergency medical care, this methodology was deemed most appropriate.

### Sampling and recruitment

Purposive sampling was used to recruit two samples from the Western Cape. The first sample included 13 (*n* = 13) academics from the Health Sciences, who had a background in community engagement and were actively involved in community engagement projects related to health or emergency medical care. There were six males and seven females in this sample. They were recruited from four higher education institutions in the Western Cape. The second sample consisted of 16 (*n* = 16) academics who were recruited specifically from departments of emergency medical care. There were nine males and four females in this sample. These academics were recruited from universities and colleges, so that the researcher could acquire a better understanding of how community engagement had been integrated into emergency medical care education. The academics from emergency medical care (EMC) were recruited through their head of department. After receiving the names of those academics willing to participate in the focus group discussion, the researcher contacted them, to brief them on their participation in a focus group interview. This sample consisted of seven females and nine males and included academics, who were involved in teaching EMC and had been involved primarily in work integrated learning.

### Data collection

Semi-structured interviews were used to gather data from sample one, related to community engagement. Initially 10 participants were recruited, but interviews continued till data saturation was reached. For the second sample, data were collected using a focus group interview. After obtaining ethics approval, data collection commenced. Access to the participants was challenging because of the coronavirus disease 2019 (COVID-19) pandemic and the lockdown regulations. Therefore, interviews were conducted when restrictions were lifted. After participants had agreed to participate in the study, appointments were scheduled with them at a time convenient to them. Before conducting the interviews, participants were required to read an information letter and sign a consent form, which indicated their willingness to participate in the study. Eight participants were interviewed in person, while five requested online interviews using the Microsoft Teams online platform. The interviews were recorded using digital recorders and field notes were taken. Each interview lasted approximately 60 min and was guided by a pre-tested interview guide. One-on-one interviews were held with sample one because they provided discipline-specific insights into community engagement as they were from various health sciences disciplines, while sample two consisted of academics in emergency medical care, therefore a focus group discussion was appropriate.

Given that 16 EMC academics had indicated their willingness to participate in the focus group interview, a focus group discussion was held. At the start of the group discussion, each participant was provisioned with a letter of information, thereby enabling them to provide informed written consent to participate. The focus group discussion was held face-to-face at the emergency medical care college in Western Cape. The focus group discussions were audio recorded and guided by a focus group schedule.

Both the interview guide and focus group guide consisted of the same questions. These questions explored how academics conceptualised community engagement; the role of higher education institutions in promoting community engagement; the challenges and strengths of community engagement; and the role of EMC education through community engagement. This approach captured deep insights and experiences whereby academics provided a rich description and layers of meaning regarding community engagement with higher education. The interview and focus group guide was piloted with five academics experienced in community engagement through one-on-one interviews. Following the pilot interview, academics suggested the use of probing questions, which were subsequently added to the interview/focus group guide.

### Data analysis

Data from the transcribed interviews and focus group discussions were coded and analysed manually, to identify initial codes. The data were first carefully read and re-read to enable the researchers to become familiar with the data. The initial emergent codes were then compared and clustered to create main categories. Themes that emerged from the sub-themes were defined through a series of reviews. This process of thematic analysis, which involved the categorisation and interpretation of the collated data, enabled interpretations of the unsaid and explicit dimensions and structures of the research phenomenon (Braun & Clarke 2019). Themes were reviewed to confirm their relevance to the research questions and through the help of an independent coder, those which were mutually supported were agreed upon.

### Trustworthiness

Steps to uphold rigour during the data collection process, included a process of consistent formatting of the interviews; audio recording and professional transcription of same (Noble & Smith 2015). The verbatim transcription of the recorded interviews and focus group discussions, enabled them to be checked for accuracy. A process log was maintained during data analysis, so that decisions made were recorded, thereby ensuring transparency. Credibility was accomplished through a thorough engagement with the literature on community engagement, repeated reading of transcripts, and member checking with participants to verify the accuracy of the collected data (Adler 2022). A detailed description of the study context was included, particularly the selection and characteristics of participants, the data collection process and the process of analysis to enable transferability of the study to another context. Co-coding of the data was conducted with a peer who is an expert in qualitative research, and the data was further validated by an expert validation committee. Members of the expert validation committee confirmed the participants’ narratives, data analysis and presentation of findings, further enhancing the study’s trustworthiness.

### Ethical considerations

Ethical clearance to conduct this study was obtained from the University of Cape Town’s Human Research Ethics Committee and ethics consent was received on 16 October 2019. The ethics waiver number is 624/2019. The 1964 Declaration of Helsinki and all amendments thereto were adhered to. Participation in the study was voluntary, and each participant received a letter of informed consent, that stipulated that their identifying details would be anonymised, and that all information gathered would remain confidential. The use of pseudonyms ensured the anonymity of participants. In addition, no names or personal identifiers appeared on any of the data sheets that were used during analysis. Participants’ involvement in the study was also strictly voluntary and they were informed that they could withdraw from the research at any time.

## Results

### Demographic information

Table 1 shows two distinct samples of academics focused on community engagement in health sciences and emergency medical care.

**TABLE 1:** Demographic profile of interviewed participants.

Sample	Number of Participants	Males	Females	Qualifications	Institution or Department	Codes
1. Health sciences academics	13	6	7	Development Studies, Medicine, Physiotherapy, Health Profession Education, Science Education	Higher Education Institutions	A – Academic e.g. A13 – Academic number 13
2. Emergency medical care academics	16	9	4	Emergency medical care, medicine	Emergency Medical Departments from Universities and Colleges	FG1 EA- focus group 1 EMC academic e.g. FG1 EA 2 – focus group 1 EMC academic number 2

EMC, emergency medical care; FG, focus group; EA, emergency care academic.

Table 2 outlines the main themes derived from the interviews.

**TABLE 2:** Themes derived from the interviews and focus group discussions.

Themes	Subthemes
1 Community engagement at the interface of emergency medical care	1.1 Void within the curriculum 1.2 Embedding community engagement in emergency medical care
2 Transformative potential of community-based teaching	2.1 Transformative learning experiences 2.2 Value of a field coordinator 2.3 Role of volunteerism
3 Strength of community-based knowledge	3.1 Honouring the wisdom of community members 3.2 Learning in community spaces

### Theme 1: Community engagement at the interface of emergency medical care

There were two subthemes, which emerged under theme one, namely the void within the curriculum and embedding community engagement in emergency medical care.

#### Subtheme 1.1: Void within the curriculum

Emergency medical care work is deeply entrenched within community spaces, thereby naturally positioning paramedic students to engage with and develop mutually beneficial partnerships with communities. Despite this one of the academics lamented that EMC education, has remained aloof from its communities saying:

‘We don’t necessarily recognise the role that we could be playing as paramedics, and emergency medical services broadly.’ (FG1 EA3)

One potential reason for this disjuncture can be attributed to its absence within the EMC curriculum:

‘Community engagement is missing in our curriculum … this should be a formal activity structured in the programme, and it can be embedded in foundations of practice in primary healthcare.’ (FG1 EA2)

Support for Boyer’s model is endorsed by one of the participants who acknowledged the indivisible relationship between emergency medical services and the communities they served, affirming the potential for emergency medical care students to become more socially responsive to societal ills. This was evidenced as follows:

‘As an EMS practitioner, you are rooted in the society which we’re trying to serve … a lot of your work is not only medical … acknowledge … all sorts of different kinds of community engagement.’ (A 9)

Other participants believed that by creating opportunities for engagement, then only could the community engagement mandate be strengthened within EMC education:

‘If health services have a strong element [*of*] community engagement, then the students would have the opportunity to participate.’ (A 13)

‘Community engagement must be driven by the university; it was curriculum driven in terms of learning outcomes. Students must gain a good understanding of health promotion and how to apply it to health …’ (A3)

#### Subtheme 1.2: Embedding community engagement in emergency medical care

The process of embedding community engagement in emergency medical care education emerged as the second subtheme under theme one. Participants shared this as follows:

‘The learning outcomes of the educational programme must represent competencies and roles that the person has to play in the real world. If you are teaching people competencies that don’t exist in the real world, then it becomes problematic.’ (A 13)

‘Curriculum needs to have a primary healthcare approach to health … your paradigm needs to shift in terms of health care in South Africa … to bring health closer to the community … The one thing in the curriculum, [*is that*], learning outcomes need to be developed that [*are*] linked to community engagement.’ (A 9)

The need for engaged, immersive pedagogies is echoed in the narratives that follow:

‘Community-oriented curriculum, meaning that you will include the concepts such as community, community health, and prevention … to expose students at [*an*] early stage of the curriculum by working with the communities … let them do a community profile of their own community. You slowly start getting them to think about those things early, then getting them to start doing, for example, the situation and analysis.’ (A 9)

‘You need to have skills in determining what the community health needs are, how you would prioritise those health needs with the community.’ (A 8)

Participants also argued the importance of interweaving notions of community and community health into the curriculum, by deliberately creating opportunities, to learn about community problems through community profiling:

‘I feel that the student experience needs to have a form of engagement, what it means to be an engaged citizen, and what social justice and social responsibility is all about. It could be service learning, experiential learning, or any credit-bearing activity, but then there is also a component of volunteerism and that’s where a social justice component comes in. This is where people realise, they need to be active citizens and that they got to find a way of ensuring that whatever experiences they have had.’ (A3)

Finally, the need for the institutionalisation of community engagement, within the university and the emergency medical care department was shared as follows:

‘Community engagement must be driven by the university; it was curriculum driven in terms of learning outcomes. Students must gain a good understanding of health promotion and how to apply it to health.’ (A 3)

### Theme 2: Transformative potential of community-based teaching

#### Subtheme 2.1: Transformative learning experiences

Boyers four forms of academic scholarship, namely the scholarship of discover, integration, application and teaching collectively, emphasises the deepening of connectedness with community, outside the academic domain. It supports the potential for acquiring greater knowledge in community spaces and for transformative learning experiences as evidenced within the narratives that follow:

‘We need [*the*] community to make sure that our students get that transformative learning experience.’ (A 12)

‘Our approach is that we basically integrate community engagement work in our teaching and learning and research.’ (A 10)

‘I think that modules that must somehow enshrine this idea … recognise the immense community-based teaching power that social participation in health systems brings when you recognise the knowledge that people in communities have, and that you shape your health interventions around that.’ (A 12)

#### Subtheme 2.2: Value of a field co-ordinator

To bridge the chasm between university and community, a field coordinator, within the emergency medical care programme, was suggested as follows:

‘We have employed a field coordinator that coordinates activities in those designated sites.’ (A 7)

‘Appointing a field coordinator, being part of the forums, and then feeding in the issues that the communities are finding challenges with, that they need some assistance with, and that gets fed through to my office or the departments via the field coordinator.’ (A 10)

Another way that students could be exposed to community related issues was through creating opportunities for students to share their experiences with them as follows:

‘One way of getting into community engagement and teaching or creating the environment for the students, is the methodology of sending various groups into the community and creating that feedback system where students can share with a group what their experience was.’ (FG1 EA 13)

‘Bringing the context of those communities back into the classroom, so that learning is relevant and also prepares the students better for practice once they graduate.’ (FG1 EA 8)

#### Subtheme 2.3: Role of volunteerism

The value of volunteerism, emerged through the voices of one of the participants, as follows:

‘In our institution we offer students the opportunity to volunteer … And then there’s opportunities where they can do it in a credit bearing form, and then … the possibilities where they develop their own community engagement activities in their communities, and in certain ways the university supports that in terms of the learning and teaching.’ (A 10)

‘I do not believe that all engagement activities at the university should be curricular based … some institutions see that it as … credit-bearing. For me, I feel that the student experience needs to have a form of engagement; what it means to be an engaged citizen, and what social justice and social responsibility is all about. It could be service learning, experiential learning, or any credit-bearing activity, but then there’s also a component of volunteerism, and that’s where a social justice component comes in. This is where people realise they need to be active citizens and that they got to find a way of ensuring that whatever experiences they have had, they must share because getting an education is a privilege.’ (A 3)

### Theme 3: Strength of community-based knowledge

The third theme that emerged from the data related to the strength of community-based knowledge.

#### Subtheme 3.1: Honouring the wisdom of community members

In the narrative that follows, community members are described as ‘street professors’, who possess a great deal of relevant information about their communities:

‘A very rich source of knowledge that was being generated from what we called “community level intelligence” or professors of the street generating this in real time, very sensitive COVID information about how people in different parts of the city were responding to the pandemic and the lockdown.’ (A 9)

This was acknowledged by other participants, who expressed that community-based knowledge, outweighed the knowledge derived within traditional didactic lectures in the classroom space. They said:

‘We wanted to give lots of lectures, yet one experience in the community would teach more than 20 lectures.’ (A 2)

Another participant reported on the value of community-based experiences, saying:

‘I know that social determinants of health is something you can learn in a book. I don’t think you really understand it until you see someone and the effect of that on their experience. We could ask them [*students*] to live with a family for the period, so that they can be in sort of immersed in the community … to expose them to the bitter truths of healthcare in our country.’ (A 8)

#### Subtheme 3.2: Learning in community spaces

Participants expressed that community-based learning experiences help to strengthen students’ understanding of the patient within their family and community context. This was described as follows:

‘That made us realise the value of community engagement …you are dealing with the person within a family, within a community … there can’t be clinical training without community engagement because you’re doing it in an experiential education model.’ (A 11)

Another participant recognised the powerful learning experience, which can emerge when students are faced with actual clinical emergencies which they have never encountered before and must cope with implementing interventions without knowledge and experience. He said as follows:

‘You put them in a situation where they are exposed to something unsettling and that is where they feel discomfort, where they’re actually in a position which gives experience … they don’t necessarily have the answers to.’ (A 11)

Other participants observed the benefits of learning about diverse cultures and the unique dynamics of certain communities:

‘The benefit of that life experience where the students obviously got to learn tolerance, based on different cultures, religion … if you’re being brought up in a different society, meaning if we have to compare [*the*] Cape Flats with a rural area, … then you need to be tolerant.’ (FG1 A 9)

‘They need to learn to adapt to the cultures, the background, as well [*as*] the lingo [*language*] of the certain communities that they enter, because what they are lacking are their soft skills … That can be a great benefit for learners.’ (FG1 EA 12)

The value and benefits of co-learning with community, which is the hallmark of engaged scholarship was further evidenced in the narrative that follows:

‘We had this learning network, with co-learning sessions three or four times a week. Some of us were going around and visiting all the different Community Action Networks … we were generating knowledge about how to build a COVID safe kitchen, how to use a kitchen as a space for doing social mobilisation awareness raising … In the lines of the kitchens, people were also handing out flyers about COVID, about how to keep themselves safe. The Health Department used a lot of the Community Action Networks’ guidance because much of what was available, was from a different setting … we worked with a range of different virologists and scientists to figure out how to make a virucidal solution from the contents of your own kitchen.’ (A 9)

The ability to secure practice-related experience was further evidenced as follows:

‘You gain life experience with different communities … you get to see, feel the patients and their circumstances … we don’t normally see.’ (FG1 EA 6)

## Discussion

The glaring void within the formal EMC curriculum, particularly regarding primary health care, underscores the traditional focus on clinical competence within health sciences, often at the expense of nurturing opportunities for students to learn about the health problems confronting local communities. Boyers’ model of scholarship recognises the interconnectedness between teaching and the application of knowledge to real-world problems, thereby contributing to preparing students to understand societal challenges. He also advocates for the use of innovative teaching methods. To achieve the integration of engagement in EMC education, academics will need to reorient their teaching and learning strategies and radically transform curricula to create enhanced opportunities for students to learn in real-time collaboratively with community partners, establishing measures for co-learning within community contexts (Bhagwan 2019).

Flogueiras et al. (2020) argued that service learning is a promising pedagogical strategy that can connect students to their communities by interweaving educational and civic goals, resulting in benefits such as positive perceptions of community engagement, the development of critical thinking and problem-solving skills, and the ability to adapt to new situations. As one academic suggested, the curriculum must create opportunities for paramedic students to be immersed in their communities during training not only to acquire competencies to become successful paramedics but also to learn about health issues confronting vulnerable and underserved populations. Boyer’s model encourages engaged teaching and learning that challenges students to be reflexive and critically engaged with real-life, contextual situations that warrant social transformation. By working towards solutions to these health issues, students can improve community health and reduce health inequities (Meurer et al. 2011).

Several factors influence health outcomes, including social determinants of health, health literacy, and access to healthcare (Fang et al. 2022). Paramedics, therefore, should be prepared not only to meet clinical disciplinary requirements but also to understand the social determinants of health and advocate for disadvantaged communities facing social and health problems. Consequently, many scholars assert that students must be connected to the community through mutually beneficial partnerships that enable them to address health issues and societal problems (Mann & Bowen 2021). These narratives further highlight the importance of service learning within the EMC curriculum. Community-based learning experiences promote knowledge transformation, moving beyond simple information transfer typical of traditional approaches (Hart, Daniels & September-Brown 2023). They emphasise that students should acquire competencies linked to real-world problems, better preparing them for practice.

Boyer (1990) proposed that the scholarship of teaching involves transforming and learning knowledge through ongoing exploration of context and pedagogy. Figueiró et al. (2022) advocate for engaged, immersive pedagogies that place students in real-world contexts, enabling them to connect complex theoretical knowledge to community challenges and prompt action. This aligns with Boyer’s model, as knowledge gained through immersion is more authentic and useful facilitating an understanding of complex community problems. By immersing students into diverse cultures, academics create pathways for extended forms of knowledge to emerge through engagement.

Experiential learning within service learning strengthens co-learning within communities (Groulx et al. 2021). Central to this approach is preparing students for the ‘real world’ and deepening their commitment to social justice issues by engaging in activities that can foster societal change (Zizka, McGunagle & Clark 2021). Currently, the disconnect between curricula and local health challenges leaves EMC graduates ill-prepared for practice and unaware of issues faced by disadvantaged populations. Revolutionising the EMC curriculum through service learning and community-based participatory research can shift focus from clinical practice to health prevention and promotion, emphasising engaged activities. Service learning that is credit-bearing introduces students to social justice and social responsibility, fostering engaged citizenship. Conscientising students about social justice helps them to understand how economic, political, and social structures perpetuate oppression and health disparities (Bhagwan 2020).

A study at a South African university concluded that authentic early community-based experiences strengthened medical students’ commitment to social responsibility (Van Wyk et al. 2016). Their knowledge and understanding of disadvantaged communities and social determinants of health improved, fostering proactive roles in local communities. Embedding social equity and justice into community-engaged curricula is crucial in preparing students to serve these communities (Bhagwan 2019). Volunteerism has been identified as a pathway for students to become more socially responsive. Ross and Kabidi (2017) support this, observing that volunteerism in paramedic education can nurture attributes essential for the profession, with some programmes requiring students to complete minimum community volunteer hours in health settings.

The participants’ narratives underscore that the community is central to learning within health sciences, serving as a rich experiential context where multiple epistemologies of knowledge coexist (Bhagwan 2019). Integrating engagement with teaching, learning, and research legitimises the community as a source of significant knowledge, dismantling traditional hierarchical notions that confine knowledge within university walls (Hart et al. 2023). Engagement fosters reciprocal relationships through practice-based, experiential, and service-learning approaches. Collaborative educational models can emerge, positioning communities as epicentres of learning, where students co-construct health interventions benefiting vulnerable populations. Engagement should be viewed as an integral part of teaching and research within higher education, resonating with Boyer’s (1990) concept of community engagement connecting universities with societal projects, fostering engaged pedagogy, community-based research, and collaborative practice.

Appointing dedicated personnel to facilitate community engagement enhances students’ immersive experiences and strengthens partnerships. Sharing real-world community experiences allows students to gain deeper insights into local social, health, economic, and environmental issues, which can be integrated into classroom discussions (Bhagwan 2019). Learner-centred, experiential, and transformative pedagogies better prepare students for future practice environments. Working respectfully and meaningfully within communities aligns with Boyer’s (1990, 2016) concerns about the scholarship of discovery, emphasising processes and meaning over outcomes. Boyer also argued that academic work must relate to the broader world beyond the university.

As one participant observed, service learning that is credit-bearing is not the only avenue for engagement; volunteer opportunities also provide valuable civic knowledge. Such experiences help students to understand societal complexities and foster a sense of social responsibility. Student volunteering is linked to higher education’s democratic purpose, nurturing social responsibility among undergraduates (Bhagwan 2019). Volunteering teaches communication, listening skills, cultural sensitivity, and awareness of diversity in a pluralistic society (Cress, Collier & Reitenauer 2023).

The traditional focus on clinical patient care often detracts from understanding broader public health challenges, a gap observed across medical and paramedic fields (Bastable et al. 2019). Engaged scholars such as Smith-Tolken and McCay (2019) emphasise community knowledge, advocating for community members to serve as mentors or co-educators, thereby enriching contextualised learning and co-creating knowledge. Recognising community partners’ wealth of knowledge is increasingly important, with collaborations involving co-teaching and curriculum input to reflect community issues (Coles-Ritchie et al. 2022).

Immersing students in communities exposes them to the realities of healthcare systems and social challenges, fostering a holistic understanding of systemic influences on health. For example, students living with families to experience hardships firsthand can better appreciate social complexities. Discovery-based learning opportunities, such as community action networks developing public health information such as the COVID-19 social awareness campaign exemplify transdisciplinary collaboration, where scientists, community members, and health professionals co-create solutions (Stokols 2006). Such partnerships demonstrate how paramedic students can collaborate within community-based participatory research projects to address local health issues.

Preparing EMC students for their roles requires immersing them in community contexts, which are more transformative than classroom experiences. These engagements increase their understanding of community health problems and socio-economic factors influencing patient care (Ohta, Ryu & Sano 2021). Community paramedicine exemplifies this approach, expanding paramedics’ roles to include primary and preventive health care and social services, thus improving access for underserved populations (Thurman et al. 2021). Beere, Votruba and Wells (2011) argued that communities should be viewed as assets sources of strength, wisdom, and knowledge from which students can learn. Ross and Kabidi (2017) concluded that engagement fosters critical skills necessary for future paramedics.

Although community-based learning may be uncomfortable, navigating unfamiliar spaces enhances students’ preparedness for practice, particularly within marginalised communities. Such experiences reveal gaps in healthcare delivery and help students to understand how socio-economic contexts shape health needs. Encounters with community members can sensitise students to patient needs and foster advocacy skills, raising awareness of issues such as alcohol abuse and mental health disorders (Bidandi 2021; Ross & Kabidi 2017).

The importance of strengthening community engagement in health sciences is especially relevant in developing countries, where access to healthcare and workforce sustainability are pressing issues (McManamny et al. 2018; Van Bewer 2017). Expanding the scope of paramedic practice to include health education, promotion, and primary care can improve community well-being. Embedding engagement into EMC curricula through service learning, outreach, and community projects is essential. Literature supports that paramedic practice can contribute to health promotion when integrated with community engagement activities (McManamny et al. 2018; Rasku et al. 2019). Although community paramedicine is still emerging, emphasising the community’s role is crucial for preparing graduates for real-world healthcare challenges (O’Meara et al. 2015; Okoh et al. 2023). The current gap in engaged strategies limits opportunities for students to work within communities through service learning, volunteerism, and participatory research.

According to Mtawa (2019), the core components of effective community engagement include student learning, curriculum transformation, community-defined priorities, and knowledge production. The commitment of academics is vital to establishing robust opportunities that enable students to understand health inequalities and local realities (Cyril et al. 2015). Such collaborative partnerships, grounded in reciprocity and mutual exchange, reflect Boyer’s (1990) view that knowledge application occurs when the work of academia relates to societal needs beyond the campus.

Participants emphasised that embedding community engagement within EMC curricula will facilitate teaching and learning that is relevant to socio-economic realities. This approach ensures that graduates are competent in addressing societal issues, fostering social justice and equity. Health promotion becomes a key outcome, with students developing a critical sense of social responsibility, informed by concepts of social justice and community health (Coelho & Menezes 2021). Advocates such as Bastable et al. (2019) call for a shift towards community-based health education, where communities serve as active teachers and repositories of knowledge, aligning with the knowledge democracy model (Benneworth et al. 2018). Universities should empower students to undertake community-based participatory research, deepening their understanding of local health issues and partnering to find community-driven solutions (Plummer et al. 2021).

Integrating engagement through service learning, outreach, volunteerism, and community-based research can create pathways for paramedic students to develop competencies that transcend traditional clinical training, fostering a holistic, socially responsible, and community-oriented approach to health sciences education. Such strategies can better prepare students for the complexities of real-world healthcare environments, ultimately contributing to healthier, more equitable communities (Dobson & Kirkpatrick 2017).

## Conclusion

Universities need to be recognised as multiple learning spaces, hence their impact on graduates should not be constrained to disciplinary expertise, but rather to nurture their personal, social and civic roles (Rutti et al. 2016). This study advances the need for emergency medical care to extend preparedness for clinical practice through conscientising students about health disparities and to working with vulnerable communities to find solutions to health problems. As Núñez (2019:97) argued ‘learning in service is a pedagogical model, which allows the university to exercise its social responsibility in the formative environment through teaching-learning processes linked with its social environment’. By actively immersing emergency medical care students into community spaces, powerful connections can emerge between theory and practice, which can strengthen student’s skills thereby building networks between the university and community.
